# Phenotypic plasticity of malignant T cells in blood and skin of a Sézary syndrome patient revealed by single cell transcriptomics

**DOI:** 10.3389/fonc.2023.1090592

**Published:** 2023-01-25

**Authors:** Lukas Peiffer, Thilo Gambichler, Terkild B. Buus, Kai Horny, Jan Gravemeyer, Frauke Furtmann, Ivelina Spassova, Linda Kubat, Laura Susok, René Stranzenbach, Nalini Srinivas, Niels Ødum, Jürgen C. Becker

**Affiliations:** ^1^ Translational Skin Cancer Research, Deutsches Konsortium für Translationale Krebsforschung (DKTK), Essen, Germany; ^2^ Deutsches Krebsforschungszentrum (DKFZ), Heidelberg, Germany; ^3^ Department of Immunology and Microbiology, University of Copenhagen, Copenhagen, Denmark; ^4^ Skin Cancer Center, Department of Dermatology, Venereology, and Allergology, Ruhr-University, Bochum, Germany; ^5^ Department of Dermatology, University of Essen, Essen, Germany

**Keywords:** scRNAseq, inflammation, reactive T cells, malignant T cells, Sézary syndrome, cutaneous T cell lymphoma

## Abstract

**Background:**

Sézary Syndrome (SS) is an aggressive leukemic variant of cutaneous T-cell lymphomas (CTCL). In SS patients, malignant T cells are circulating through the blood and cause erythroderma.

**Objective:**

To compare the transcriptome of single cells in blood and skin samples from a patient with advanced SS.

**Methods:**

We utilized combined single cell RNA and T-cell receptor (TCR) sequencing (scRNA-seq).

**Results:**

We scrutinized the malignant T cells in blood and skin in an unbiased manner without pre-sorting of cells. We observed different phenotypes of the same monoclonal malignant T-cell population, confirmed by TCR sequencing and inferred copy number variation analysis. Malignant T cells present in the circulating blood expressed genes resembling central memory T cells such as *CCR7*, *IL7R* and *CD27*. In the skin, we detected two major malignant T-cell populations: One subpopulation was closely related to the malignant T cells from the blood, while the other subpopulation expressed genes reminiscent of skin resident effector memory T cells including *GZMB* and *NKG7*. Pseudotime analysis indicated crucial transcriptomic changes in the transition of malignant T cells between blood and skin. These changes included the differential regulation of *TXNIP*, a putative tumor suppressor in CTCL, and the adaptation to the hypoxic conditions in the skin. Tumor cell proliferation in the skin was supported by stimulating interactions between myeloid cells and malignant T cells.

**Conclusions:**

Using scRNA-seq we detected a high degree of functional heterogeneity within the malignant T-cell population in SS and highlighted crucial differences between SS cells in blood and skin.

## Introduction

Cutaneous T-cell lymphoma (CTCL) comprise a group of non-Hodgkin lymphomas exhibiting chronically inflamed skin lesions. The most prevalent forms are mycosis fungoides (MF) and the more aggressive leukemic variant Sézary syndrome (SS) (1). In MF patients, malignant T cells are mainly confined to the skin and resemble a skin effector memory T-cell phenotype. In SS patients, however, malignant T cells are also circulating through the blood and resemble a central memory T-cell phenotype, expressing markers such as of CD197 (*CCR7*), CD27 and CD62L (*SELL*) (2). High-throughput TCR sequencing confirmed that malignant T cells are derived from a monoclonal T-cell population (3). However, identification of malignant T cells based on surface marker expression is notoriously difficult and variable strategies have been applied to isolate malignant T cells (4). Mostly, malignant SS cells were characterized by the lack of markers such as CD26 and CD7 (5).

Recent studies utilizing single cell RNA sequencing have provided new insights into the heterogeneous expression of marker genes in malignant T-cell populations in SS. For instance, Buus et al. (6) identified different subpopulations within the malignant T-cell clone based on a highly heterogeneous expression of typical marker genes. Interestingly, these subpopulations are differently affected by histone deacetylase inhibitor (HADCi) treatment. Besides the heterogeneity on a single cell level, treatment of SS is additionally complicated by great inter-patient differences. Several somatic copy number alterations have been demonstrated in SS patients, but a single common cancer driving mutation have not been identified and personalized therapies appear to be warranted (7).

In this study, we compared the transcriptome of single cells in blood and skin samples from an advanced SS patient. Our unbiased approach without pre-sorting enabled us to identify distinct phenotypes of the same malignant T-cell clone within the blood and skin of the same patient.

## Materials and methods

### Patient

Study material including a skin biopsies and blood was obtained from a patient suffering from advanced SS. This patient was recruited at the Skin Cancer Center, Department of Dermatology, Ruhr-University Bochum, Germany. The ethics committee of the University Duisburg-Essen approved the project (18-8230-BO) and it was conducted in accordance with the declaration of Helsinki.

### Sample preparation for single cell RNA sequencing

A fresh tissue biopsy from an erythrodermic lesion was immediately processed after excision. The generation of a single cell suspension was performed according to the tumor dissociation protocol by Miltenyi (Bergisch Gladbach, Germany). Briefly, the tissue biopsy was cut into small cubes of 2 mm and then minced with the gentleMACS program “h_tumor_01” and two times “h_tumor_02” with 30 min incubation time after each mince. During the incubation the sample was digested with an enzyme cocktail mix consisting of 200 µl Enzyme H, 100 µl Enzyme R and 25 µl Enzyme A (catalogue #130-095-929 Miltenyi Biotec, Bergisch Gladbach, Germany). The cell suspension was reconstituted in PBS with 0.05% BSA and washed three times, before passing through a 100 µm cell strainer. Subsequently, the cells were processed with the Single Cell 5’ Library & Gel Bead Kit v1.1 (catalogue #1000165, 10xGenomics, Pleasanton, CA, USA) according to the manufacturer’s protocol. Processing of the blood sample comprised isolation of peripheral blood mononuclear cells by gradient centrifugation and reconstitution in PBS with 0.05% BSA before continuing with the 10xGenomics protocol. As input for the protocol, 20,000 cells from the skin tissue and 8,000 cells from the blood were used, both at a concentration of 1000 cells/µl. To correct for doublets, a hashing procedure was performed for each sample. Before the start of the 10xGenomics protocol, the single cell suspension was divided into three equal pools and incubated with a unique TotalSeqC hashing antibody (1 µg for 1-2 million cells) (Biolegend, San Diego, CA, USA) for 30 min at 4°C. Afterwards, the cell suspensions were washed three times and pooled in equal amounts before continuing with the 10xGenomics protocol.

The resulting gene expression and surface antibody libraries were sequenced at the DKFZ Genomics and Proteomics core facility on a NovaSeq6000. 8,313 cells were recovered from the tissue sample and 3,748 from the blood sample. T-cell receptor libraries were sequenced on a NextSeq 550 platform with Paired-End 150bp Mid-Output.

### Data analysis

The cellranger pipeline (v3.0, 10xGenomics) was used with default settings respective for gene expression and TCR libraries to process the sequencing data. Initial analyses were performed using the Seurat package (v3.2.2) in R software (v.3.40). Samples were merged with the “merge” command by Seurat and subsequently processed as described in the following. Quality metrics for filtering were chosen as following: mitochondrial gene count <12%, UMI count >500, gene count >200 and housekeeper gene count >40 (8). Gene expression counts were log-normalized and the 2000 most variable genes were selected for calculation of principal components (PC). When calculating PCs, G2M and S-phase scores of the cells were used to regress out cell cycle effects for clustering (9). To depict the cells by uniform manifold approximation and projection (UMAP), dimensional reduction and clustering was performed by using the top 20 PCs (resolution of 0.5). Differential gene expression was calculated by Wilcoxon Rank Sum Test (log fold change (FC) > |0.25|, adjusted P value < 0.05). Finally, clonotype information was added to the meta data of the Seurat object based on the “filtered_contig_annotations” file.

The inferCNV package (https://github.com/boradinstitute/inferCNV) by the Trinity CTAT project was used to infer large chromosomal copy number variations from the sequenced single cell RNA. Reactive T cells were used as a reference. To infer the activity of prominent transcription factors (TF) from single cell data, DoRothEA (discriminant regulon expression analysis) was used, a database containing a curated collection of transcription factors and their transcriptional targets (regulon) (10). Only TF-target interactions with a confidence level of A, B or C were considered. The activity of a TF was then calculated based the mRNA expression level of its targets by VIPER (virtual inference of protein activity by enriched regulon analysis) (11).

### Analysis of ligand-receptor interactions

Interactions between T-cell subsets and dendritic cells were assessed as described by Roider et al. (12) Briefly, ligand-receptor interactions were taken from the CellPhoneDB database (13) and the mean interaction score was calculated by multiplying the mean expression of ligand in cell type A with the mean expression of receptor in cell type B. Significance was determined by “calculating the proportion of permuted interaction scores that were by hazard higher than the actual interaction score. To determine which interactions were most relevant across different samples, we calculated the mean interaction scores and combined the different P values using the Fisher’s method.” In the end, P values were corrected by utilization of the Benjamini–Hochberg method.

## Results

### Patients’ history

**Figure 1:**
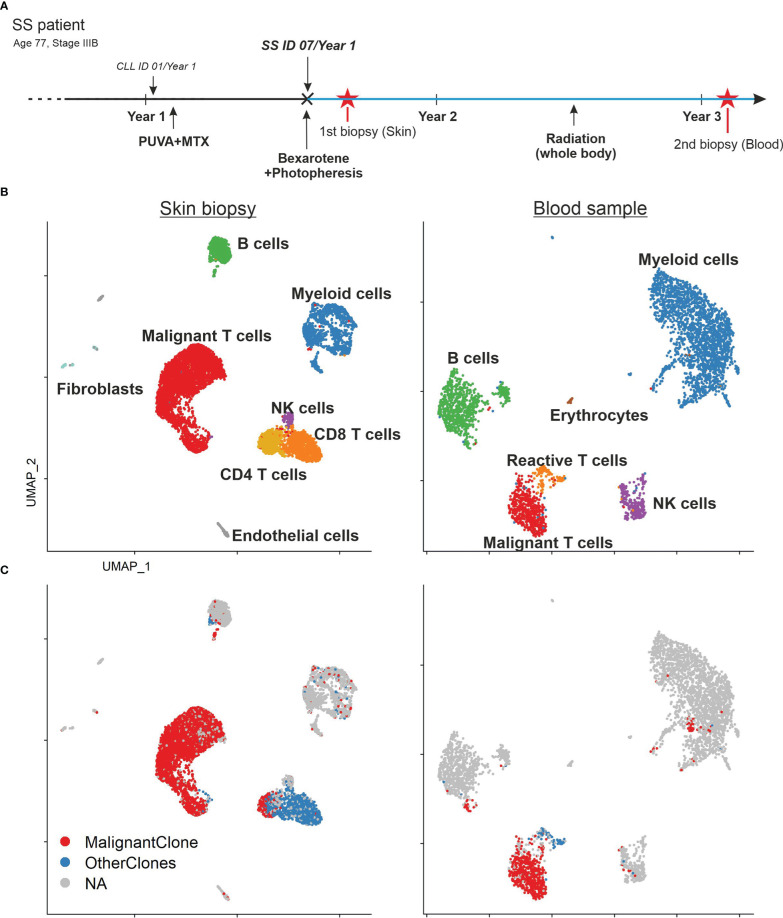
Patient’s history and sample composition. **(A)** Time line of the patient’s clinical history. Red asterisks mark the time point of the biopsies. The blue lines indicate the ongoing bexarotene and extracorporeal photopheresis (ECP) therapy. The patient was initially misdiagnosed with chronic lymphatic leukemia (CLL) and psoriasis before the diagnosis of SS. **(B)** UMAP of all cells within the skin biopsy (left) and blood sample (right) according to the similarity of their transcriptome and annotated by their cell type. **(C)** UMAP of all cells within the skin biopsy (left) and blood sample (right) color coded by TCR clonotype annotation. MalignantClone includes all cells with the TCR composed of TCRα: CAVTGARLMF; TCRβ: CSASTGGAYTEAFF. OtherClones includes all other TCRs.

The clinical history of the patient is depicted in **Figure 1A**. The presented patient was initially (January 2019) misdiagnosed with chronic lymphocytic leukemia (CLL) and psoriasis. At this time, she was treated by her dermatologist with PUVA and methotrexate for 2 months, but stopped because of adverse events. In July 2019 she was re-diagnosed with SS (stage IVAI) (**Suppl. Figure 1**) (1). Upon occurrence of circulating SS cells, the patient’s therapy included bexarotene as well as extracorporeal photopheresis (ECP), which were well tolerated and resulted in a clinical improvement. Because of worsening of her condition whole-body radiotherapy was performed in June 2020. Combined bexarotene and ECP therapy was maintained. However, her condition was progressive and mogamulizumab was initiated in August 2022. After the first cycle, she developed tumor lysis syndrome and bacteriemia. In September 2022, she died from septic shock.

Despite the common origin in one malignant T-cell clone, malignant T cells in SS patients often express a high diversity of surface markers (6). This tumor plasticity is especially visible in different tissue compartments (14). In this study, we wanted to address this heterogeneity of SS tumor cells by an unbiased sampling approach. We performed combined single cell RNA and TCR sequencing of unsorted cells from a skin biopsy and PBMCs from the whole blood of the same SS patient. Thus, we acquired the transcriptome of 7,531 cells from the skin and 3,902 cells from the blood (**Figure 1B**). Cells were manually annotated according to classical markers for the respective cell type (15). Among these cells, we found 5,762 T cells expressing *CD3E* and *CD3G*. Application of the TCR data revealed the presence of a malignant T-cell population consisting of overall 3,813 T cells with the common monoclonal TCR (TCRα: CAVTGARLMF; TCRβ: CSASTGGAYTEAFF) in both, the skin as well as the blood sample (**Figure 1C**). Additionally, the single cell TCR sequencing revealed a monoclonal B cell population with IGH: CAKDRGGTFDAFDIW and IGK: CQQYYSYPRTF comprising 83% of all B cells in the skin biopsy and 88% of all B cells in the blood sample.

### Malignant T cells in SS exhibit variable phenotypes

**Figure 2:**
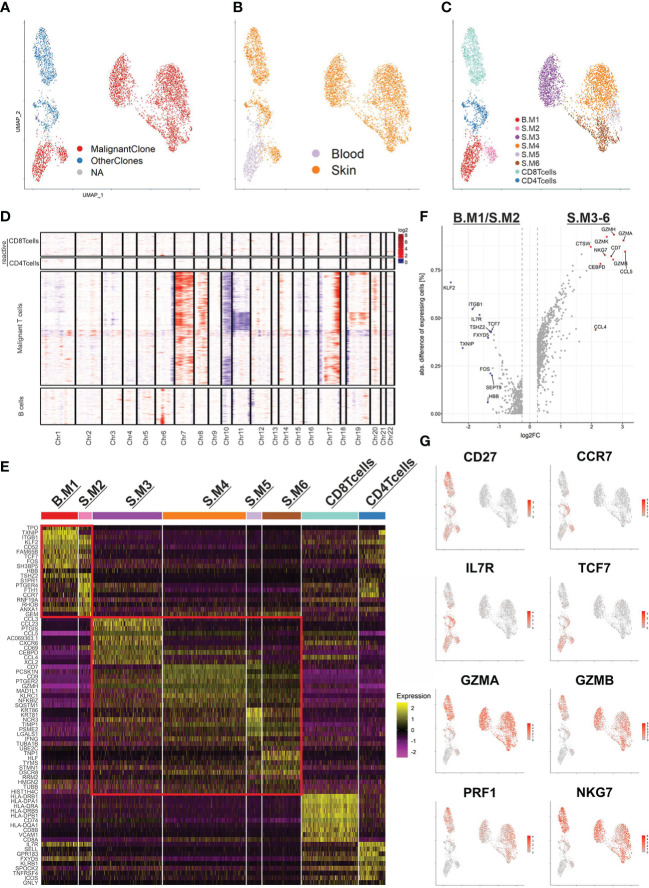
The malignant T-cell clone and its subpopulations. **(A)** UMAP of all T cells (5,453) from the blood and skin biopsy color coded by TCR clonotype annotation. MalignantClone includes all cells with the TCR composed of TCRα: CAVTGARLMF; TCRβ: CSASTGGAYTEAFF. OtherClones includes all other TCRs. **(B, C)** UMAP of all T cells highlighted by sample origin **(B)** and cluster annotation **(C)**. **(D)** CNV analysis using reactive T cells used as reference. Chromosomal gains are marked in red and chromosomal losses in blue. The x-axis shows the affected chromosomes. **(E)** Heat map of the top 10 differentially expressed genes of each annotated cluster. Two red rectangles are marking the malignant T cells with memory phenotype (upper) and cytotoxic phenotype (lower). **(F)** Volcano plot of the differentially expressed genes between the two major subpopulations of the malignant T-cell clone. **(G)** Feature plots highlighting the most discriminative genes from **(E, F)**.

In order to scrutinize the malignant T-cell clone, we merged both samples, subset our data set for T cells only and applied the TCR sequencing data (**Figure 2A, B**). The graph-based clustering approach by Seurat revealed 8 visually distinct clusters (**Figure 2C**). According to canonical markers, we identified a cluster of reactive CD4^+^ T cells (*CD27*, *SELL* (CD62L), *CCR7*, *TCF7*) originating from skin and blood and one cluster with reactive CD8^+^ T cells (*CD8B*, *GZMB*, *NKG7,CD27*) originating from the skin. The malignant T-cell population was split into 6 different clusters (Blood/Skin malignant T-cell cluster 1-6 (B./S.M1-6)). While one cluster comprised all the malignant T cells from the blood (B.M1), the other 5 clusters comprised the malignant T cells from the skin (S.M2-6).

Furthermore, we applied inferCNV to reveal large copy number variations of the malignant T-cell population using reactive T cells as reference (**Figure 2D**). The malignant T-cell clusters indeed harbored common gains and losses. Amplifications in chromosome 7 (*CARD11*), 8q and 17q, as well as characteristic losses in chromosome 10 (*ZEB1*, *PTEN*) and 17p (*TP53*) have been described in SS before (7). Additionally, a loss of chromosome 6q (*FOXO3*) and 19p (*SBNO2*, *ZBTB7A*) have been observed. Of note, the CNV analysis revealed a subclonal population of malignant T-cells with additional losses in chromosome 11 and gains in chromosome 19. However, since this population is strongly exhibiting cell cycle related gene expression, the differing CNV profile might be an artifact rooting in the RNA inference (**Suppl. Figure 2**). Interestingly, the monoclonal B-cell population did not share genetic alterations with the malignant T-cell clone and only possessed an amplification of chromosome 6p (MHCI and MHCII).

Subsequently, we examined the top 10 differentially expressed genes (DEG) of each malignant T-cell cluster (**Figure 2E**). Already visible in the UMAP and represented in the heat map, the malignant T-cell clusters could be separated into two larger populations. Strikingly, one population consisting of the malignant T-cell clusters 1 and 2 (B.M1/S.M2) were closely resembling memory T cells, expressing *CD27*, *CCR7*, *IL7R*, *TCF7*. The other population consisting of S.M3-6 were closely resembling cytotoxic T-cells, expressing effector genes such as *GZMA*, *GZMB*, *PRF1* and *NKG7* (**Figure 2F, G**). Of note, S.M3 additionally expressed high levels of chemokines such as *CCL3*, *CCL4* and *CCL5*, which regulate recruitment and differentiation of T-cells (**Figure 2E**). In summary, our data demonstrate a high heterogeneity of the malignant T-cell clone and implicate a phenotype type switch of malignant T-cells, depending on the tissue compartment.

**Figure 3:**
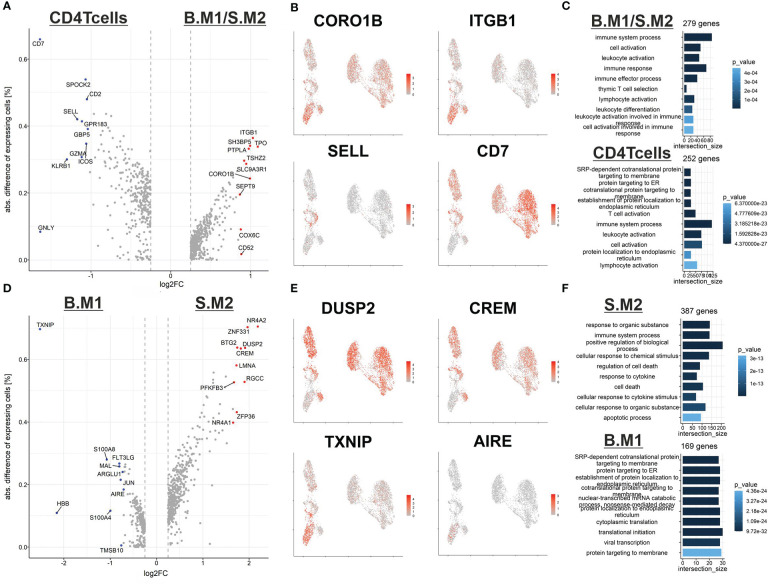
Differences between reactive and malignant T cells from blood and skin. **(A)** Volcano plot of differentially expressed genes between reactive CD4 central memory T cells (left) and malignant T cells with a central memory phenotype (right). **(B)** Feature plot highlighting genes from the DEG analysis from **(A)**. **(C)** GO-term analysis of the DEG between reactive central memory T cells (bottom) and malignant T cells with a central memory phenotype (top). **(D)** Volcano plot of differentially expressed genes between malignant T cells with a central memory phenotype from the blood (left) and malignant T cells with a central memory phenotype from the skin (right). **(E)** Feature plot highlighting genes from the DEG analysis from **(D)**. **(F)** GO-term analysis of the DEG between reactive central memory T cells (bottom) and malignant T cells with a central memory phenotype (top).

To gain a better understanding of T-cell malignancy, we investigated the differences between reactive CD4^+^ T cells and the memory like malignant T cells (B.M1/S.M2) (**Figure 3A, B**). Of note, the malignant T-cells expressed higher levels of *CORO1B*, which might be important for survival of naïve T-cells and T-cell trafficking similar to *CORO1A*. Additionally, higher expression of *ITGB1* (CD29) was shown, which might be important for the transition from or into the skin (16). Despite the expression of many genes typical for central memory T cells, the malignant T cells did not express *SELL* (CD62L). *CD7* expression was downregulated in the memory like malignant T cells, but was expressed in the cytotoxic malignant T cells. This might explain the variable expression of CD7 found in SS patients in previous studies. Gene ontology (GO) term analysis revealed a similar terminology for both populations centering on T-cell activation (**Figure 3C**).

Next, we compared the memory like malignant T cells from the blood (B.M1) against the skin (S.M2) (**Figure 3D, E**). We identified an upregulation of genes such as *DUSP2* and *CREM* in malignant T cells in the skin, which might be early signs of counter regulative mechanisms of T-cell activation. Additionally, we observed a downregulation of the autoimmune regulator *AIRE*, whose role in peripheral immune regulation is still under investigation, and the tumor suppressor *TXNIP* (17). Interestingly, *in vitro* experiments demonstrated low expression of TXNIP in malignant T cells compared to non-malignant T cells. The reduced expression is mediated by different mechanisms including epigenetic regulation *via* the methyltransferase enhancer of zeste homolog 2 (EZH2) or overexpression of miR-106b (18). Of note, induced expression of TXNIP strongly inhibited malignant T-cell proliferation, indicating that TXNIP acts as a potential tumor suppressor in CTCL (18). GO-term analysis revealed that the malignant T cells are more prone to cell death and might need to adapt to the specific environment of the skin (**Figure 3F**). However, it might also be a consequence of the stress from dissociation of the tissue, which induces an apoptotic signature in the cells from the skin.

### Pseudotime analysis demonstrates transcriptomic changes in malignant T cells involved in the transition between blood and skin

**Figure 4:**
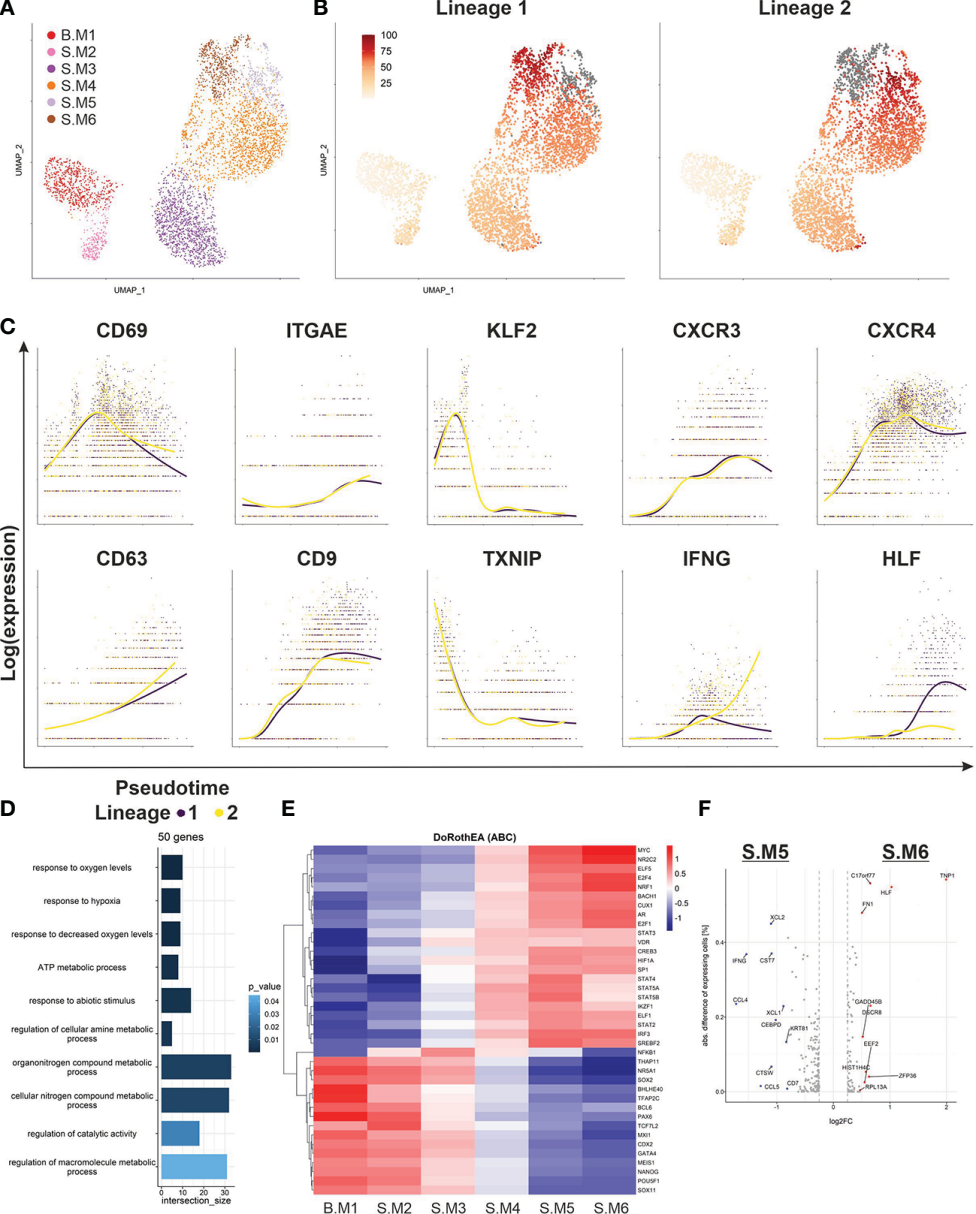
Pseudotime analysis of malignant T-cell population. **(A)** UMAP of all malignant T cells from blood and skin biopsy annotated according to their transcriptomic subtype. **(B)** Pseudotime trajectories visualized by color shaded from yellow to red for the malignant T cell clone as derived from Slingshot and ending in either S.M5 (Lineage 1) or S.M6 (Lineage 2) depicted in grey. **(C)** Depiction of marker and functional gene expression of the malignant T-cell clone along the pseudotime trajectories. **(D)** GO-term analysis of the top 50 genes, which were differentially expressed between the beginning and end of pseudotime identified by TradeSeq. **(E)** Heat map showing the regulon activity in all malignant T-cell clusters of the most variable TFs identified by VIPER and based on DoRothEA. **(F)** Volcano plot of differentially expressed genes between the two endings of the respective pseudotime trajectories, S.M5 and S.M6.

In order to understand the mechanisms driving the phenotype switch of malignant T cells, we subset our data set for malignant T cells only and applied Slingshot to infer pseudotime (**Figure 4A**). Clusters were annotated as similar as possible to the previous analysis based on their DEG. Notably, Slingshot identified two trajectories, starting in memory like malignant T cells from the blood (B.M1), differentiating into cytotoxic malignant T cells in the skin and finally separating into the clusters S.M5 and S.M6 (**Figure 4B**). Next, we investigated marker genes, which were closely related to pseudotime (**Figure 4C**). Malignant T cells in the skin upregulated skin retention markers *CD69* and *ITGAE* (CD103) and downregulated *KLF2*, a transcription factor important for T-cell trafficking. A downregulation of *KLF2*, also leads to higher levels of chemokine receptors (19). The increased expression of the tetraspanins *CD9* and *CD63* may represent important co-stimulatory signaling of malignant T cells in SS patients (20). Furthermore, we observed a downregulation of *TXNIP*, corresponding to the increased activation levels of the T cells in the skin, confirming the recent findings of a deficient *TXNIP* expression due to epigenetic repression in malignant T cells (18). *TXNIP* inhibits proliferation of malignant T cells and the multifunctional protein thioredoxin (TRX), which has been shown to protect cells from oxidative stress (21). Thus, downregulation of *TXNIP* may be a crucial part of the adaption to low oxygen levels and enhanced proliferation of malignant T cells in the skin. This adaptation process is also reflected by the GO-term analysis of the top 50 genes, which show the highest differences between start and end of the pseudotime (**Figure 4D**). Additionally, a prediction of transcription factor activities by DoRothEA and VIPER revealed increased activity of *HIF1α* in the clusters towards the end of pseudotime (**Figure 4E**). We could also observe an increase in the activity of signal transducer and activator of transcription (STAT) genes, as well as *IRF3*, which is important for T-cell effector functions. In contrast, clusters at the beginning of pseudotime showed higher activity of transcription factors crucial for maintaining pluripotency such as *SOX2* and *NANOG* (22). Finally, we compared the endings of the two pseudotime lineages, S.M5 and S.M6 (**Figure 4F**). Interestingly, while S.M5 strongly expressed effector genes such as *IFNG*, S.M6 expressed *HLF*, a transcription factor involved in hematopoietic stem cell properties. Taken together, pseudotime analysis demonstrated subclonal divergence of malignant T cells with a memory phenotype in blood and a cytotoxic phenotype in the skin, where malignant T cells possibly retain a self-renewable population and adapt to the low oxygen environment. However, the calculated pseudotime cannot accurately predict if the malignant T cells migrate from blood to skin or vice versa.

### Cellular interactions in SS skin lesion

**Figure 5:**
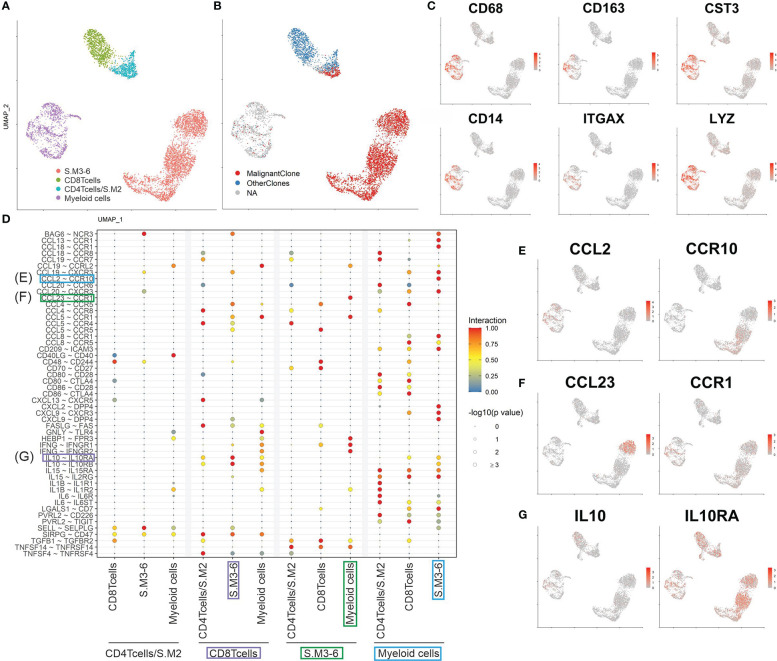
Cell interactions in SS skin lesion. **(A, B)** UMAP of all T cells and myeloid cells from the skin biopsy color coded by cluster and TCR clonotype annotation. **(C)** Feature plots of classical markers for myeloid cells. **(D)** Plot showing the most significant ligand-receptor interactions. The size of the circle reflects the negative log_10_ of the adjusted *P* values, which were calculated by a permutation test (Methods). The interaction scores were calculated by multiplying the mean expression of the ligand with the mean expression of the respective receptor and normalized within the cell type’s interactions. (E to G) Feature plots of possible CCL2/CCR19 **(E)**, CCL23/CCR1 **(F)** and IL10/IL10RA **(G)** interactions between cells present in the skin.

Evidence is cumulating that myeloid cells are fueling inflammation in CTCL skin lesions (23). Therefore, we analyzed interactions of T cells and myeloid cells derived from the skin biopsy of the SS patient studied here (**Figures 5A, B**). Myeloid cells were identified by classical marker expression as seen in **Figure 5C**. Subsequently, ligand-receptor interactions were predicted using CellphoneDB (**Figure 5D**) (13). We revealed an intricate network of interactions, where malignant T cells and myeloid cells are stimulating each other and thus driving inflammation. For instance, myeloid cells are recruiting CCR10 expressing T cells to the skin by secretion of CCL2 (**Figure 5E**) (24). On the other hand, malignant T cells influence migration of CCR1 expressing myeloid cells *via* the secretion of CCL5 and CCL23 (**Figure 5F**) (25). Moreover, myeloid cells promote tumor growth by secretion of pro-inflammatory cytokines such as *IL1b*, *IL6* and *IL15*, which have been shown to promote inflammation and tumor growth (26). Especially, IL15 has been shown to promote tumor progression of CTCL (27). Furthermore, myeloid cells possibly suppress anti-tumor responses by secretion of IL10 and interaction *via* CD80/CD86 with CTLA-4 on reactive CD8^+^ T cells (28). In turn, reactive CD8^+^ T cells are also expressing *IL10*, which might represent a defense mechanism against the malignant T cells (**Figure 5G**). In summary, myeloid cells indeed seem to play a role in the formation of a tumor-promoting environment by recruiting malignant T cells, stimulating a pro-inflammatory environment and suppressing anti-tumor responses.

## Discussion

The high inter- and intra-patient heterogeneity of CTCL poses a major challenge for diagnosis and successful treatment of the disease (29). Investigation of this heterogeneity within a patient is now possible in an unprecedented manner by single cell RNA sequencing. Simultaneous sequencing of the TCR enables the identification and characterization of the malignant T-cell clone. In this study, we scrutinized the malignant T-cell clone in blood and skin from an advanced SS patient. Notably, albeit the clonal origin of the malignant T-cell clone, evident by the expression of a monoclonal TCR, we detected the existence of two major phenotypes. It has been suggested that MF cells are derived from skin resident effector memory T cells and SS cells are derived from central memory T cells (2). Here, malignant T cells in the blood were expressing markers typical for central memory T cells such as *TCF7*, *CCR7* and *IL7R*. A similar population of T cells was also found in the skin biopsy; however, the larger fraction of malignant T cells in the skin was expressing effector molecules such as *PRF1*, *GZMB* and *NKG7*. This cytotoxic phenotype demonstrates the phenotypic plasticity of SS cells (4). To our knowledge, the existence of two highly different phenotypes of the same malignant T-cell clone in one patient has not been reported before. An explanation for this could be that most studies so far investigated SS cells either only from the blood or the skin or that technological limitation allowed only to analyze a narrow marker set when obtaining matching samples from blood and skin. A recent single cell analysis comparing malignant T cells from lymph node, skin and blood in MF did show a similar plasticity of the malignant T-cell clone (30). However, the cytotoxic phenotype was much less pronounced.

Furthermore, we observed expression of *CD7* and *DPP4* (CD26) in the malignant T cells in the skin, but not in the blood. These surface marker genes have often been used to identify tumor burden of SS patients in blood by FACS analysis. Nevertheless, their expression has been reported to be variable, which might be partly explained by our findings (31). Moreover, Buus et al. (6) reported a high variability of surface marker expression in SS cells on a single cell level, stating that neither CD26 nor CD7 was sufficient to identify the malignant T-cell population in all patients. Using multiparameter flow cytometry Nadjidh et al. recently confirmed the heterogeneity of Sézary cell subpopulations within and between patients (32).

The observed plasticity of the malignant T-cell clone might be a necessity to be able to adapt to the different conditions in blood and skin. Our pseudotime analysis suggests that the change in the microenvironment results in a phenotype switch of the malignant T cells. However, the direction of migration is still unclear. In contrast to the direction suggested by the pseudotime algorithm in this study, recent genomic analysis revealed a UV-signature in CTCL cells, indicating a migration from skin to blood (33). A phenotype switch has also been reported as response to bexarotene therapy (34). Bexarotene promotes Th1 responses and might have caused the expansion of the cytotoxic phenotype in the skin of the patient investigated in this study (34). Another reason for the highly cytotoxic phenotype in skin lesions could be the presence of external pathogens like *Staphylococcus aureus* (35). Indeed, elimination of skin-colonizing bacteria using antibiotic treatment was sufficient to relieve symptoms of advanced CTCL patients (36).

Notably, towards the end of pseudotime the malignant T-cell clone split into two subpopulations, one expressing *IFNG* and one with possible stem cell properties (*HLF*). This might indicate that malignant T cells do not recirculate to the blood, but maintain a subpopulation of self-renewable cells in the skin. During a longer course of the disease, this would add to the intra-patient heterogeneity with varying malignant subpopulations in different skin lesions and blood. However, to confirm this hypothesis, the genomic profiles of malignant T-cell populations from different skin lesions of the same patient would need to be compared. If they show the exact same genomic alterations, it is unlikely that they developed individually. Additionally, we could observe a subpopulation expressing high amounts of chemokines, possibly responsible for attracting more immune cells to the skin lesion and drive inflammation. This confirms previous reports of the heterogeneity of the malignant T-cell population on a single cell level and the identification of essential subpopulations might implicate new strategies for targeted therapy (6).

Furthermore, we could demonstrate that SS cells are adapting to lower oxygen levels in the skin. In this regard, the downregulation of *TXNIP* might be a crucial step for the skin transition of the malignant T-cell clone. Due to the fact that induced TXNIP expression inhibits the proliferation of malignant T cells, it has been suggested as possible tumor suppressor in CTCL (18). Indeed, treatment with 2-deoxyglucose (2DG) is a potent inducer of TXNIP and has been shown to be effective against lymphoma cells (37). Moreover, the HIF-1α regulon was more active in malignant T cells in the skin late in pseudotime. Treatment of HIF-1α has been shown to be effective in CTCL in a xenograft tumor mouse model (38). Hence, the adaptation to the skin microenvironment might offer new targets for SS therapies. Despite the highly cytotoxic phenotype in the skin and previous reports of the expression of T-cell exhaustion markers in CTCL cells, we could not detect significant expression in the malignant T cells (39). However, reactive CD8^+^ T cells indeed showed features of exhausted T cells, which indicate a suppressed anti-tumor response. Our interaction analysis suggests that myeloid cells might suppress the anti-tumor response by engaging CTLA-4 on CD8^+^ reactive T cells and additionally secrete a variety of chemokines, which possibly aid in the stimulation and recruitment of malignant T cells.

In summary, we demonstrate that the single cell transcriptome of SS cells, which are present in circulating blood and skin, reveals a new level of tumor heterogeneity in SS, thereby confirming the potential of scRNAseq to improve our understanding of CTCL (40–42). The existence of two distinct phenotypes of the same malignant monoclonal T-cell population driven by the tumor microenvironment represents a new finding, extending previous reports of phenotype plasticity. Indeed, the presence of different tumor cell phenotypes in the skin and blood might also explain the different response to treatments (e.g. mogamulizumab, alemtuzumab) in these tissue compartments (43, 44). CTCL lesions have often been reported to be dominated by a Th2 type inflammation (30), but the presence of cytotoxic malignant T cells shows that immune modulating therapies may change that and have to be closely monitored. Nevertheless, our results warrant to be confirmed in larger cohorts to evaluate if the existence of two phenotypes of the same malignant T-cell clone is a common feature in SS and how therapies can be amended for that.

## Data availability statement

The datasets presented in this article are not readily available because of ethical/privacy restrictions. Requests to access the datasets should be directed to the corresponding author.

## Ethics statement

The studies involving human participants were reviewed and approved by University Duisburg-Essen (18 8230 BO). The patients/participants provided their written informed consent to participate in this study. Written informed consent was obtained from the individual(s) for the publication of any potentially identifiable images or data included in this article.

## Author contributions

Conception and design: LP, TG, RS, and JB. Acquisition of material and treatment of patient: TG, RS, and LS. Bioinformatic analyses: LP, KH and JG. Experimental procedures: LP, FF, IS, LK, and NS. Analysis and interpretation of the data: LP, TB, IS, LK, TG, NS, NO and JB. Writing and review of the manuscript: LP, TG, TB, NO and JB. All authors contributed to the article and approved the submitted version.

## Supplementary material

Supplementary Figure 1 Clinical images of a patient with Sezary syndrome (SS). **(A)** Erythroderma is shown also including the hyperkeratotic soles **(B)** and palms **(C)**. At this time, routine histopathology as well as immunohistochemistry studies of skin samples, blood smears clearly showing presence of Sézary cells, the detection of monoclonal T-cell receptor rearrangements, highly increased CD4/CD8 ratio in peripheral blood, substantial loss of CD7 and CD26 expression on CD3+/CD4+ cells, and the clinical presentation was consistent with SS in stage IVA1.

Supplementary Figure 2 Inferred CNV profile of cycling malignant T cells. **(A)** UMAP based on non-regressed clustering of all T cells colored by their cell cycle gene expression. **(B)** Inferred CNVs of malignant T cells separated in cells in G1-phase and cycling cells in S- and G2M-phase.
