# Supplementary figures and images for: Phenotypic plasticity of malignant T cells in blood and skin of a Sézary syndrome patient revealed by single cell transcriptomics
Source: Front Oncol. 2023 Jan 25;13:1090592. doi: 10.3389/fonc.2023.1090592 (PMC9905421; doi:10.3389/fonc.2023.1090592)

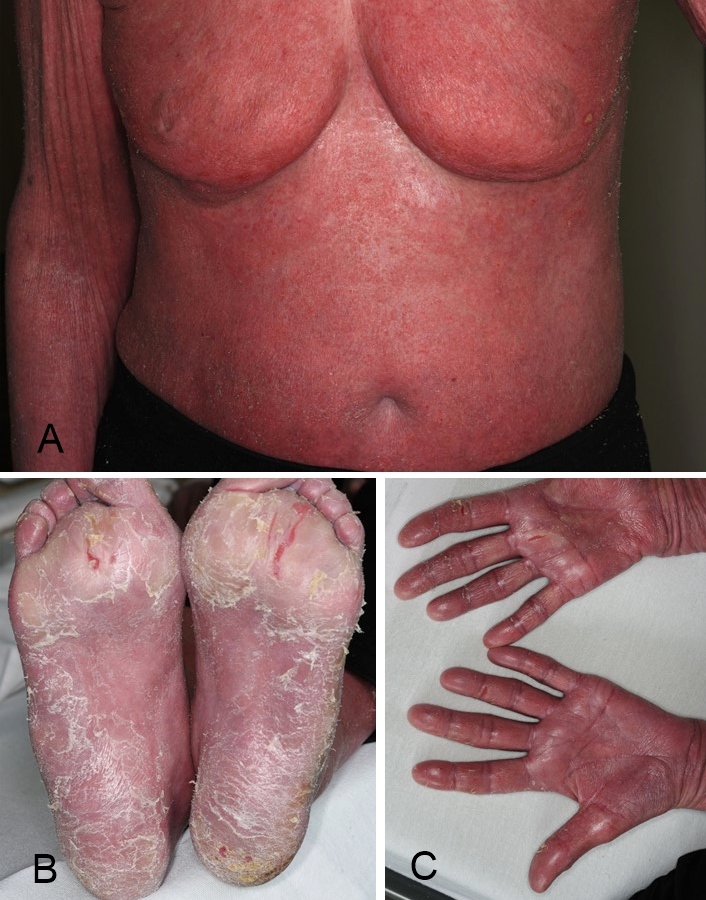

Supplement: Supplementary Figure 1 — Clinical images of a patient with Sezary syndrome (SS). (A) Erythroderma is shown also including the hyperkeratotic soles (B) and palms (C). At this time, routine histopathology as well as immunohistochemistry studies of skin samples, blood smears clearly showing presence of Sézary cells, the detection of monoclonal T-cell receptor rearrangements, highly increased CD4/CD8 ratio in peripheral blood, substantial loss of CD7 and CD26 expression on CD3+/CD4+ cells, and the clinical presentation was consistent with SS in stage IVA1. [file Image_1.jpeg]

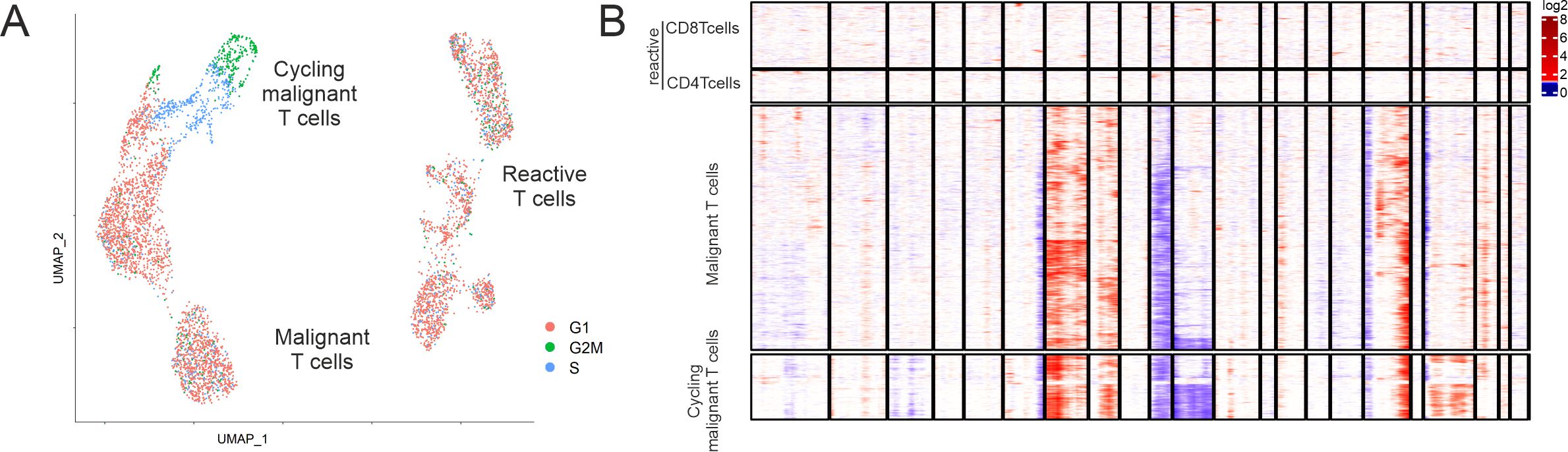

Supplement: Supplementary Figure 2 — Inferred CNV profile of cycling malignant T cells. (A) UMAP based on non-regressed clustering of all T cells colored by their cell cycle gene expression. (B) Inferred CNVs of malignant T cells separated in cells in G1-phase and cycling cells in S- and G2M-phase. [file Image_2.jpeg]
